# Prognostic significance of eighth edition TNM stage criteria in combined small-cell lung cancer

**DOI:** 10.3389/fonc.2023.1018288

**Published:** 2023-03-08

**Authors:** Ziran Zhao, Yibo Gao, Fengwei Tan, Qi Xue, Shugeng Gao, Jie He

**Affiliations:** Thoracic Surgery Department, National Cancer Center/National Clinical Research Center for Cancer/Cancer Hospital, Chinese Academy of Medical Sciences and Peking Union Medical College, Beijing, China

**Keywords:** combined small cell lung cancer (c-SCLC), 8th TNM stage criteria, prognostic significance, SEER database, surgery

## Abstract

**Objectives:**

This study aimed to evaluate the prognostic significance of the eighth edition TNM stage criteria in patients with combined small-cell lung cancer (C-SCLC) on a population level.

**Methods:**

Using the Surveillance, Epidemiology, and End Results (SEER) database, patients diagnosed with C-SCLC (histology code 8245) between the years 2004 and 2015 were identified. We performed a Kaplan–Meier analysis and used the multivariable cox regression proportional hazards model to obtain prognostic overall survival estimates for each group of patients.

**Results:**

A total of 477 patients diagnosed with C-SCLC were identified. The T, N, M, TNM, and combined TNM stage status of the eighth edition were all significant prognostic factors for patients’ overall survivals, with the best discrimination identified in the combined stages. Surgery was also found to be a prognostic factor (HR =1.95, 95%CI =1.49-2.56, p<0.01) for patients with C-SCLC.

**Conclusions:**

The combined eighth edition of the TNM staging criteria shows reliable prognostic significance in patients with C-SCLC. Moreover, surgery might be significant for improving the patients’ prognosis.

## Introduction

Lung cancer is one of the most prevalent malignancies and the leading cause of cancer-related mortality worldwide (1). Combined small-cell lung cancer (C-SCLC) is a relatively uncommon subset of lung neuroendocrine cancer (NEC), defined as small-cell lung cancer (SCLC; the most lethal subgroup) combined with additional components of non-small-cell lung cancer (NSCLC) (2). The NSCLC components could be different or mixed, without amount restrictions, unlike the minimum 10% of components for large-cell neuroendocrine carcinoma. The SCLC practice guidelines were used for the diagnosis and treatment of patients with C-SCLC, but aside from the NSCLC components, even the cell origins and overall survivals might be different (3).

The most recent eighth edition of the TNM cancer classification criteria for lung cancer was published in 2016, based on the International Association for the Study of Lung Cancer (IASLC) database (4). Compared to the seventh edition, there are significant refinements in the T and M components, decreased size boundaries, and more accurate metastases sites (5–7). Although these refinements were analyzed from the data of NSCLC patients, they were validated in SCLC in the IASLC database, National Cancer Data Base, and Chinese cohorts (8). However, to our knowledge, prognostic assessments of C-SCLC, another subset of NEC, have not yet been performed on a population level.

The purpose of our study was to evaluate the prognostic significance of the eighth edition TNM stage criteria in patients with C-SCLC by using the Surveillance, Epidemiology, and End Results (SEER) database on a population level (9). Moreover, we aimed to analyze the demographic and clinical treatments factors associated with survival outcomes. This might allow us to optimize the management procedures for these patients and finally improve the prognosis.

## Methods

We searched for and collected data of patients from the Surveillance, Epidemiology, and End Results (SEER) database, which provides cancer treatment and survival information covering approximately 48.0 percent of the U.S. population (9). Based on the November 2020 submission, patients diagnosed with C-SCLC (histology code 8245) between the years 2004 and 2015 were identified. For the anonymized and public data, written informed consent and additional institutional review board approval were waived.

The patients included in this study were those who were reported from hospitals and clinics, were diagnosed with positive histology, had only one primary tumor, had a defined follow-up period, and who did not have missing staging information. Patients who died of other causes and represented unknown demographic and clinical variables were excluded. We determined the clinical TNM stages according to the eighth edition criteria. Because the numbers of T1a(n=11), IA1(n=4), IIA(n=7), and IIIC(n=8) stage tumors were small, we combined stages T1a and T1b (denoted as T1a&b), IIA, and IIB (denoted as II), as well as IIIB and IIIC (denoted as IIIB&C). Furthermore, stages IA1, IA2, and IA3 were grouped together as stage I for analysis.

We calculated the absolute numbers and percentages of each group of patients with C-SCLC. The median overall survivals were identified for each group. A Kaplan–Meier analysis was performed to obtain the prognostic overall survival estimates for each group of patients. The multivariable cox regression proportional hazards model was used to evaluate the prognostic significance of the eighth edition TNM stage criteria in C-SCLC. Statistical analysis was performed by the SPSS version 26.0(IBM) and PRISM version 9.3(GraphPad).

## Results

A cohort of 477 patients diagnosed with C-SCLC between the years 2004 and 2015 were included in this study, including 296 (62.05%) patients aged between 40 and 70 years old, 247 (51.78%) male patients, 250 (52.41%) married patients, and 397 (83.23%) white patients. Regarding the primary sites of the tumors, 6.08%, 62.05%, 4.19%, and 23.69% were located in the main bronchus and the upper, middle, and lower lobe, respectively, while 3.98% were not located in any specific lung sites. Most patients (213, 44.65%) were in stage IV, 36.89% in stage IVA, and 7.76% in stage IVB. Meanwhile, 11.95%, 5.66%, 9.01%, 11.74%, and 16.98% of the patients were in stages IA, IB, II, IIIA, and IIIB&C, respectively. In total, 350 (73.38%) cases received chemotherapy, while 245 (51.36%) and 146 (30.61%) patients received radiation and surgery, respectively. For combination treatments, 138 (28.93%) patients underwent both surgery and chemotherapy, while 103 (21.59%) patients underwent both surgery and radiation (**Table 1**).

**Table 1:** Demographic variables, median survivals, and multivariable cox regression for patients with C-SCLC.

Characteristics	Patients No.	Percentage	Median Survivals (months, 95% CI)	Hazard Ration	95% CI	*P* Value
Age						
40-70	296	62.05%	26.00 (22.11-29.89)	1	NA	NA
>70	181	37.95%	18.25 (14.99-21.51)	1.30	1.06-1.60	0.01
Sex						
Female	230	48.22%	24.55 (20.55-28.55)	1	NA	NA
Male	247	51.78%	21.67 (17.93-25.41)	NA	NA	NA
Marital Status						
Married	250	52.41%	23.31 (19.69-26.93)	1	NA	NA
Unmarried	227	47.59%	22.78 (18.64-26.93)	NA	NA	NA
Race, ethnicity						
White	397	83.23%	22.23 (19.35-25.10)	1	NA	NA
Others	80	16.77%	27.19 (19.28-35.09)	NA	NA	NA
Primary Site						
Main bronchus	29	6.08%	11.66 (7.50-15.81)	1	NA	NA
Upper lobe	296	62.05%	25.67 (22.03-29.30)	NA	NA	NA
Middle lobe	20	4.19%	25.00 (4.45-45.55)	NA	NA	NA
Lower lobe	113	23.69%	19.6.0 (14.69-24.51)	NA	NA	NA
Lung, NOS	19	3.98%	18.37 (5.05-31.69)	NA	NA	NA

CI, confidence interval; NA, not applicable.

**Figure 1:**
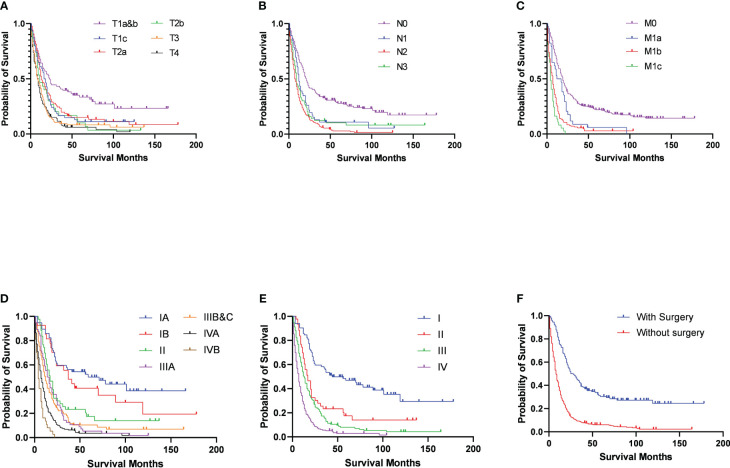
Survival outcomes in patients with C-SCLC stratified by eighth edition TNM stage criteria **(A)** T-stage; **(B)** N-stage; **(C)** M-stage; **(D)** TNM-stage; **(E)** combined TNM-stage) and surgery **(F)**.

After the determination of the eighth edition TNM stages, it was found that the T stage, N stage, M stage, TNM stage, and combined TNM stage statuses were all significant prognostic factors for the patients’ overall survivals (**Figures 1A-E**). With the increasing stage status, decreasing overall survivals were found in nearly all different stage status groups. In the groups of the T1a&b, T1c, T2a, T2b, T3, and T4 stages, the median overall survivals were 38.52, 24.93, 27.79, 22.80, 16.63, and 14.12 months, respectively. Only one exception was found in the overall survivals between the N2 stage (13.20 months) and N3 stage (19.83 months), increasing overall survivals with the stages. The N0 stage group conferred a median overall survival of 36.81 months, compared with those for the N1 stage (20.15 months). The M0 stage group had a median overall survival of 32.78 months and declined to 17.94 months in the M1a stage group, 10.61 months in the M1b stage, and 6.19 months in the M1c stage groups. For the TNM stage status groups, the IA and IB stage groups had almost the same median overall survivals of 52.67 and 52.59 months, respectively, which then declined steeply to 30.14 months in the II stage group. Similarly, the median overall survivals of the IIIA and IIIB&C stage groups were almost the same, at 21.64 and 21.28 months, respectively. In the IVA and IVB stage groups, the median overall survivals were the shortest, at 12.02 and 6.19 months, respectively. Moreover, the median overall survivals of the combined TNM stage status groups were 52.64, 30.14, 21.43, and 11.01 months from stages I to IV, respectively. Analysis of other clinical treatment different status groups gave the maximum difference in surgery status groups, and it was found that the overall survival of patients who received surgery was 41.58 months, compared with those without surgery (**Table 2**).

**Table 2:** TNM stage characteristics, median overall survivals, and multivariable cox regression for patients with C-SCLC.

Characteristics	Patients No.	Percentage	Median Survivals (months, 95% CI)	Hazard Ration	95% CI	*P* Value
T-stage						
T1a&b	81	16.98%	38.52 (29.90-47.14)	1	NA	NA
T1c	54	11.32%	24.93 (16.96-32.90)	1.40	0.95-2.06	0.09
T2a	86	18.03%	27.79 (20.18-35.40)	1.46	1.03-2.07	0.04
T2b	30	6.29%	22.8 (12.19-33.41)	1.52	0.96-2.39	0.07
T3	107	22.43%	16.63 (11.91-21.34)	1.55	1.10-2.18	0.01
T4	119	24.95%	14.12 (10.77-17.47)	1.54	1.10-2.16	0.01
N-stage						
N0	168	35.22%	36.81 (30.98-42.64)	1	NA	NA
N1	46	9.64%	20.15 (12.87-27.43)	1.33	0.92-1.90	0.13
N2	200	41.93%	13.20 (10.93-15.46)	1.66	1.29-2.14	<0.01
N3	63	13.21%	19.83 (11.96-27.69)	1.03	0.73-1.45	0.88
M-stage						
M0	264	55.35%	32.78 (28.44-37.13)	1	NA	NA
M1a	34	7.13%	17.94 (11.52-24.36)	1.05	0.72-1.54	0.80
M1b	142	29.77%	10.61 (8.25-12.96)	2.07	1.64-2.63	<0.01
M1c	37	7.76%	6.19 (4.39-7.98)	3.08	2.11-4.49	<0.01
TNM-stage						
IA	57	11.95%	52.67 (42.32-63.02)	1	NA	NA
IB	27	5.66%	52.59 (33.63-71.56)	1.05	0.54-2.07	0.88
II	43	9.01%	30.14 (19.72-40.56)	2.12	1.20-3.73	0.01
IIIA	56	11.74%	21.64 (15.38-27.91)	1.79	1.03-3.12	0.04
IIIB&C	81	16.98%	21.28 (15.04-27.53)	1.63	0.88-3.03	0.12
IVA	176	36.89%	12.02 (9.74-14.30)	2.78	1.64-4.74	<0.01
IVB	37	7.76%	6.19 (4.39-7.98)	4.95	2.60-9.41	<0.01

CI, confidence interval; NA, not applicable.

For the multivariable cox regression analysis in patients with C-SCLC, T-stage (T2a, hazard ratio [HR] =1.46, 95%CI =1.03-2.07, p=0.04; T3, HR =1.55, 95%CI =1.10-2.18, p=0.01; T4, HR =1.54, 95%CI =1.10-2.16, p=0.01; referred to T1a&b), N-stage (N2, HR =1.66, 95%CI =1.29-2.14, p<0.01; referred to N0), M-stage (M1b, HR =2.07, 95%CI =1.64-2.63, p<0.01; M1c, HR =3.08, 95%CI =2.11-4.49, p<0.01 referred to M0), TNM-stage (II, HR =2.12, 95%CI =1.20-3.73, p=0.01; IIIA, HR=1.79, 95%CI =1.03-3.12, p=0.04; IVA, HR =2.78, 95%CI =1.64-4.74, p<0.01; IVB, HR=4.95, 95%CI =2.60-9.41, p<0.01; referred to IA), and the combined TNM stage (II, HR =2.07, 95%CI =1.26-3.40, p<0.01; IV, HR=2.84, 95%CI =1.84-4.39, p<0.01; referred to IA) were significantly associated with bigger HRs of patients with C-SCLC. Moreover, ages older than 70 years (HR =1.30, 95%CI =1.06-1.60, p=0.01) compared to ages between 40 and 70 years, and cases without surgery (HR =1.95, 95%CI =1.49-2.56, p<0.01) compared to those with surgery, were significantly associated with bigger HRs of patients with C-SCLC (**Figure 1F**). There were bigger HRs, but without statistical significance in the other stage status groups. Likely, T1c (HR =1.40, 95%CI =0.95-2.06, p=0.09) and T2b (HR =1.53, 95%CI =0.96-2.39, p=0.07) were bigger HRs, but not significantly associated with those referred to T1a&b (**Table 3**).

**Table 3:** Combined TNM stage and treatment characteristics, median overall survivals, and multivariable cox regression for patients with C-SCLC.

Characteristics	Patients No.	Percentage	Median Survivals (months, 95% CI)	Hazard Ration	95% CI	*P* Value
Combined TNM-stage						
I	84	17.61%	52.64 (43.57-61.71)	1	NA	NA
II	43	9.01%	30.14 (19.72-40.56)	2.07	1.26-3.40	<0.01
III	137	28.72%	21.43 (16.99-25.87)	1.58	0.99-2.52	0.06
IV	213	44.65%	11.01 (9.08-12.94)	2.84	1.84-4.39	<0.01
Surgery						
Yes	146	30.61%	41.58 (35.23-47.92)	1	NA	NA
No	331	69.39%	14.89 (12.61-17.17)	1.95	1.49-2.56	<0.01
Chemotherapy						
Yes	350	73.38%	22.97 (19.95-25.98)	1	NA	NA
No	127	26.62%	23.31 (17.24-29.39)	NA	NA	NA
Radiation						
Yes	245	51.36%	21.49 (18.15-24.84)	1	NA	NA
No	232	48.64%	24.71 (20.34-29.08)	NA	NA	NA
Surgery and Chemotherapy						
Yes	138	28.93%	28.85 (23.51-34.19)	1	NA	NA
No	339	71.07%	20.70 (17.56-23.85)	NA	NA	NA
Surgery and Radiation						
Yes	103	21.59%	26.11 (20.36-31.85)	1	NA	NA
No	374	78.41%	22.22 (19.11-25.32)	NA	NA	NA

CI, confidence interval; NA, not applicable.

## Discussion

In this study, we described and validated the prognostic significance of the eighth edition TNM stage criteria in C-SCLC using population-based data from the SEER plus database. Approximately half of the population of the USA was encompassed in the SEER database; the prognostic significance of the eighth edition TNM stage criteria in C-SCLC might be generalizable and better in representing the population experience. Moreover, this huge and relatively uniform number of patients with C-SCLC and the adequate follow-up information enhance the power of our study and improve the ability to characterize reliable key factors associated with the prognosis.

To our knowledge, this is the retrospective cohort study with the largest sample size that focuses on the prognostic significance of the new edition of the TNM stage criteria in C-SCLC. We confirmed the survival outcomes separation and the progressive decline of overall survival by advancing the stage groups of the new edition. In the new edition, the size boundaries between the groups were decreased, such as stage T2, which includes tumor diameters of 3 to 5 cm instead of 3 to 7 cm in the seventh edition criteria, resulting in better distinguishing performance in the T-stage subcategories (10). Furthermore, the metastases subcategory was refined, while the previous staging categorized any metastatic case to stage IV (11, 12). Our results showed that M1c had a significantly worse prognosis than M1b, consolidating better distinguishing performance in the metastases subcategory refinement by the eighth edition. However, the categories of the N stage saw nearly no update from the previous criteria, while the N2 and N3 groups showed overlap and no significant differences in overall survivals. This finding may present a limitation on the application of the new TNM staging criteria, particularly in the N stages, which should be tailored in the upcoming staging system for patients with C-SCLC.

It was thought that surgery therapy is mostly suitable for the patients with early-stage C-SCLC (13). Our study showed the all-different-staged patients without surgery were significantly associated with a bigger HR and were referred to surgery. The median overall survival time in the surgery group was much longer than that in the non-surgery group. For the chemotherapy and radiation therapy status groups, the median overall survival time became longer when the therapy combined with surgery. This was consistent with the findings of previous studies, which showed that the 5-year overall survival rate in the C-SCLC surgery group was 48.9%, which is much higher than the 36.6% recorded in the non-surgery group (14). For all C-SCLC patients, even those with extended disease, it was considered to be potentially helpful to receive surgery for better prognosis. This was consistent with the finding in the NSCLC patients, as well as in some late-stage malignancies such as breast cancer and colorectal cancer (15–18). Therefore, surgery might be significant for improving the prognosis, and the surgical indication should be broader in patients with C-SCLC. Radiation was proved to improve the prognosis only in patients with stage III C-SCLC, and chemotherapy was significantly associated with better prognosis in both stage III and IV patients. This appears reasonable, and it is almost the same with NSCLC patients. It might be necessary for future studies to investigate patients who would be suitable for surgery and what kind of surgery would be suitable for them.

Despite the sufficient evidence and reasonable arguments, there are still some limitations that should be considered in our study. The first limitation is that our study was a retrospective cohort analysis, with retrospective research limitations such as selection bias. In addition, the lack of the component information of C-SCLC—whether small-cell lung cancer combined with adenocarcinoma or the others—might influence the outcomes of the patients and thus produce bias. Finally, the number of patients was limited in some of the subgroups, which were poorly represented. This might limit the overall survival estimation in these groups. A larger cohort of patients and detailed information are needed to validate the observed survival difference.

## Conclusion

In conclusion, our study shows that the combined eighth edition of the TNM staging criteria has reliable prognostic significance in patients with C-SCLC, and surgery might be significant for improving the prognosis. While the new TNM stages subcategories do not have good applicability and discrimination from their adjacent groups, further study is needed for the reclassification of the upcoming TNM staging and in patients with C-SCLC.

## Data availability statement

Publicly available datasets were analyzed in this study. This data can be found here: www.seer.cancer.gov.

## Author contributions

Conception and design: ZZ, SG, and JH; collection and assembly of data: ZZ and YG; data analysis and interpretation: all authors; manuscript writing: ZZ, FT, and QX; final approval of manuscript: all authors; accountability for all aspects of the work: all authors. All authors contributed to the article and approved the submitted version.
